# Reform in Medical Education

**Published:** 1863-11

**Authors:** 


The following very excellent remarks and suggestions upon the subject of medical education, we copy from the editorial of the New York Medical Times, because they are equally as applicable to the dental as the medical student. We can not let pass any opportunity of giving true and impressive ideas upon this subject, and we ask a careful reading of the following.—Ed.

Reform in Medical Education.—The medical session of the year has been inaugurated by the opening of the medical schools throughout the country. We have reluctantly omitted this year to devote a number of the "Medical Times" to a full statement of the faculties, regulations, etc., of the different schools. Although we deem it important to make an annual exhibit to the profession of the number and regulations of the Educational bodies, there have been so few changes since our last publication of a Students' Number that we may safely refer to the number of last year for any desired information. But we can not allow the occasion to pass without a word of exhortation on that most trite of all subjects—medical education.

If there is one question which should pre-eminently interest medical men it is the rank which the profession takes among other professions, and in the community. Every one is naturally jealous of the position of his caste in society. He rightly feels that his own individual character is involved in the public estimate of his associates. Whatever affects the reputation of the whole in a large degree affects the individual. The medical profession of this country has exhibited a most commendable interest in regard to its social position, and has striven with great energy to improve it. With just discrimination it has apprehended the sources of evil, and has endeavored to remove them. But, although these attempts have failed to accomplish all, they have accomplished much, and every true friend of medical reform should take courage, and redouble his efforts.

The first great defect in our system of medical education is the want of a sifting process in the selection of those who are about to commence the study of medicine. The door is thrown widely open, and every one is admitted to enter. No discrimination is made between the intellectual and the imbecile, the educated and illiterate, the moral and immoral. This grand defect must necessarily invite to our ranks all the floating mass of incompetent young men who wish to enter a learned profession. They have but to take their seats in the class rooms of the colleges to be accredited students of medicine. No question is asked as to their natural or acquired qualifications for the study. They may pass through their entire term without being asked a question as to the progress they are making. But it may be said that they are caught at last when they enter the "Green Room." This ordeal, however, is too often the merest farce. The student who has studied three years, has attended two full courses of lectures, the last of which was at "this college," and has punctually paid all the fees, is caught by no such drag-net as an examination for graduation. He slips through its coarse meshes without a wriggle. The first step in a reform of our system of medical education must be the preliminary examination of the student. Before he is allowed to matriculate, he must be examined as to his intellectual fitness for the study of an abstruse and all-embracing science, as to his educational preparation for the higher departments of scientific investigation, and as to his moral fitness for high social position. This reform is reasonable, is just, and is absolutely necessary to the elevation of the profession. We may evade this discrimination of the applicants for admission to the ranks of the profession still longer, but we can not by any other process compensate for the adulteration which it contains. The schools may enlarge their courses, and increase their facilities for study to any extent, but they can never make qualified medical men of the mentally incompetent, or thorough students of the illiterate.

Important as is this reform to the dignity, scientific character, and social advancement of the profession, and much as the subject has been agitated, we regret that no school has yet had the courage to adopt the plan of the preliminary examination of applicants for admission. The desire to have large classes, a most unworthy ambition of itself, governs the policy of every school in the country. This competition is carried to a most disastrous extent. Instead of excluding incompetent students, they freely admit them; nay more, in some cases they even place a price upon their heads. If the profession desires reform, it must demand it at the hands of the schools. Let the American Medical Association refuse to recognize a Medical College that does not institute such examinations, and faithfully carry them out.

The second defect in our system of medical education is the want of proper facilities in our schools for giving clinical as well as didactic instruction. By clinical instruction we mean what the term implies, viz., instruction at the bedside of the sick. The student should be personally brought to appreciate symptoms, to diagnosticate and prognosticate diseases, and to employ remedial agencies. He should not only become proficient in the principles of his profession, but he should also become expert in practice. It is possible for every competent student to attain to this degree of qualification, and before he graduates. The profession ought to ask nothing less of the schools. But in order to this the school must be attached to the hospital. Herein lies the basis of true medical teaching. Didactic and clinical medicine must be taught in the same course, on the same day, and by the same teacher. The precept must be at once reduced to practice if we would fix it ineradicably in the mind, and render it easy of correct application in future. It is a false theory which directs the student to complete his education before he attends the hospital. The golden opportunity is thus lost to impress the lesson of science. Besides, of the three or four thousand medical students who graduate annually from our various colleges, how few can become internes of hospitals, even if they seek such positions. The great majority must go forth from the lecture room to the stern duties of life, unskilled in the art which they are called to practise. The remedy is at hand. Every school must be required to connect itself with a hospital and make thorough clinical instruction a part of its course of study.

A third defect in our system of medical education is a short course of lectures to the students in mass, without any special adaptation of instruction to the wants of the juniors or seniors. The same lesson is taught indiscriminately to the beginner and to the student about to graduate. A radical change is required in this respect. The course of instruction should be extended over the three years, and the class be divided and subdivided according to the studies pursued. Every student would then pursue his studies systematically, under the immediate tuition of the master.

And, finally, there should be an examining board, independent of the schools, which should have the sole power of deciding the question of graduation of each individual student. Such a board would exercise a most salutary influence, being a check to all excesses on the part of the colleges in endeavoring to graduate large classes.

We have hinted at only a few of the more prominent evils in our system of education, and suggested the proper reforms. No one can doubt that they are of vital importance to the well-being of our profession. The reform rests with the schools, but the body of the profession must apply the stimulus. The former will not move unless impelled by the latter. Every medical man who has any regard for the character and position of his profession should bring his influence to bear through some powerful medium, as an association, directly upon the schools.—*American Med. Times.*

